# Innovative techniques in the treatment of penile strangulation: a case report

**DOI:** 10.1097/MS9.0000000000002105

**Published:** 2024-04-29

**Authors:** Wael Gazzah, Mahdy Mzoughi, Rayen Lahouar, Yassine Najjai, Sahbi Naouar, Braiek Salem

**Affiliations:** Department of Urology, University of Sousse, Faculty of medicine, Ibn El Jazzar Hospital, Kairouan, Tunisia

**Keywords:** case report, emergency medical services, foreign bodies, genital diseases, penile strangulation, surgical instruments

## Abstract

**Introduction and importance:**

Penile strangulation is a medical emergency characterized by the encirclement of the penis by an external object, resulting in circulatory compromise.

**Case presentation:**

A 35-year-old male presented with penile pain and urinary obstruction due to the inability to remove the ring. Upon examination, the ring was firmly lodged at the base of the penis, causing significant swelling and discoloration in the distal region.

**Interventions and outcomes:**

Initial attempts to cut the ring using standard tools were unsuccessful, leading to the engagement of a rescue team equipped with an air cutter. The cutting procedure, complicated by the ring’s thickness and hardness and the significant edema, took ~90 min. Safety measures, including the use of a surgical brain spatula and forceps, were employed to protect the penile skin from damage during the operation.

**Relevance and impact:**

This case underscores the necessity for timely intervention in penile strangulation cases and highlights the effectiveness of collaboration with specialized rescue teams equipped with appropriate cutting tools. It also emphasizes the importance of safety considerations when employing nonmedical devices in medical emergencies. The patient experienced a favorable outcome, with significant improvement in swelling and discoloration postprocedure, and no complications during follow-up. This report contributes to the limited but crucial literature on managing penile strangulation, particularly regarding the methods and timeframes for safely removing constricting objects.

## Introduction

Highlights

- Detailed case report of penile strangulation caused by a metal ring, emphasizing emergency medical intervention.
- Utilization of specialized cutting tools, including an air cutter, to successfully remove the constricting object.
- Collaborative effort between medical staff and a rescue team, illustrating interdisciplinary approach in emergency medicine.
- Analysis of the procedure’s challenges, such as the hardness of the metal and the patient’s significant edema.
- Discussion of different cutting techniques and tools, referencing previous reports and comparing their efficacies.

Penile strangulation is an urgent medical condition characterized by the encirclement of the penis by an external object that results in a circulatory compromise. This phenomenon often leads to significant swelling and edema in the distal portion of the penis^1^. Although rare, penile strangulation requires prompt and effective medical intervention due to the severe complications that can arise^2^.

The epidemiology of penile strangulation is not well documented, primarily due to the rarity and often private nature of these incidents. However, cases reported in the literature indicate that such events, while rare, are significant for their potential for severe tissue damage and the complex challenges they present in emergency medical care^1^.

The present case report delineates an instance of penile strangulation caused by a metallic ring, which was successfully removed with the collaboration of a specialized rescue team. This case is notable for the robust nature of the constricting object and the complexities involved in its removal, highlighting the importance of multidisciplinary teamwork and innovation in emergency treatment techniques. This report provides valuable information on the methods and time frames necessary for the safe removal of constricting objects, offering critical guidance for clinicians who encounter similar cases of penile strangulation.

### Case presentation

A 35-year-old man presented primary symptoms of penile discomfort and urinary obstruction attributable to an unsuccessful attempt to remove a metal ring encircling his penis.

The medical history was insignificant, and he was employed as a mechanic in the automotive repair industry. The event transpired at ~2 am, when the patient intended to use the metal ring for sexual stimulation and discovered that it was irreversibly attached, resulting in urinary challenges and penile pain. Subsequently, he sought emergency medical attention and arrived at our hospital at 9:43 am the same day.

**Figure 1:**
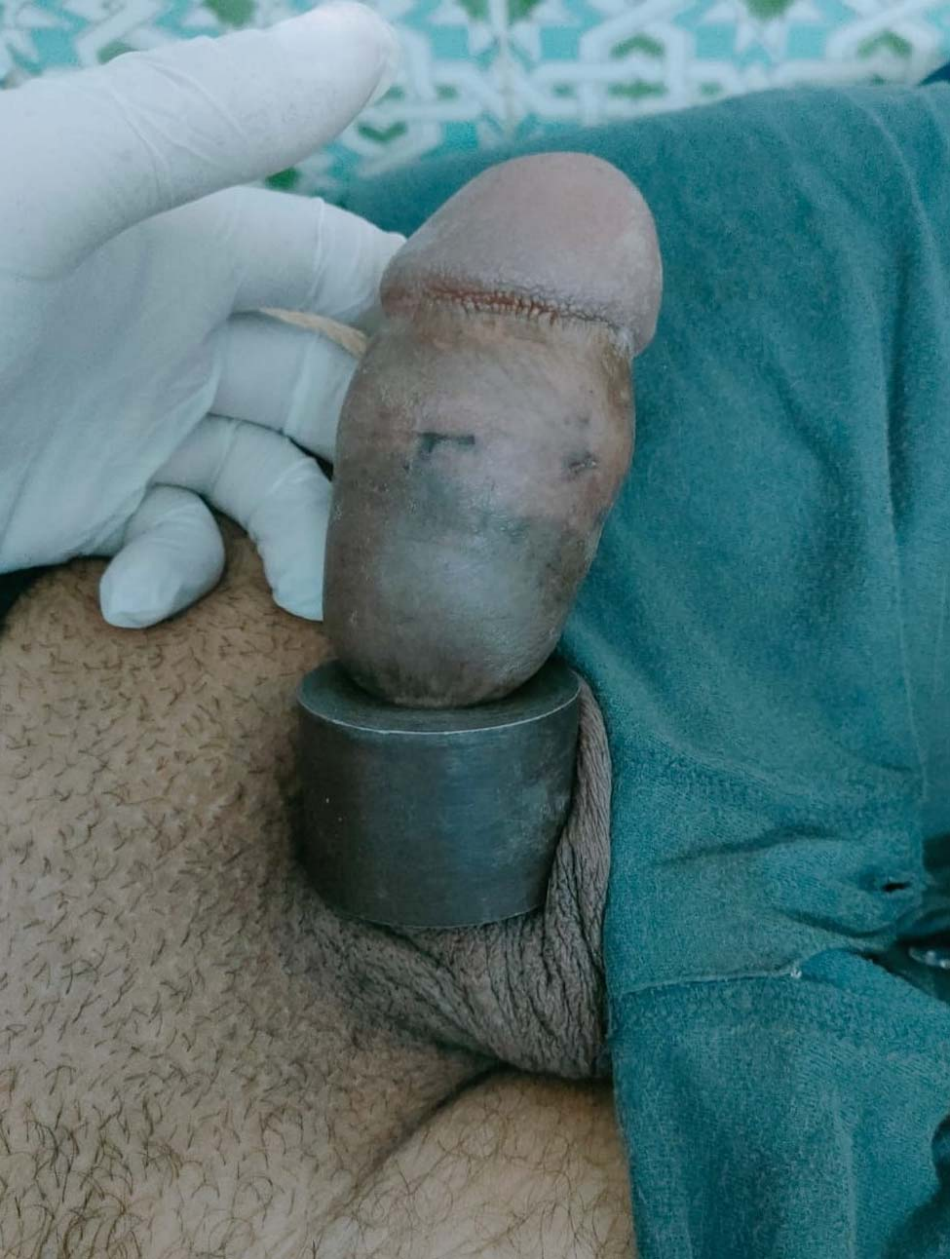
‘Initial presentation of penile strangulation’. This image shows the severity of the strangulation with the encircling metal ring measuring 30 mm in diameter and 20 mm in thickness, highlighting the resulting swelling and discoloration of the penile tissue beyond the construction site.

During the initial assessment, a metallic ring measuring ~30 mm in diameter and 20 mm in thickness was found fixed at the base of the penis. The distal segment of the penis, which extended beyond the constricted region, showed significant swelling and presented a dark red to blue discoloration (Fig. 1). In particular, the patient retained the ability to urinate in small volumes, suggesting the absence of apparent urethral damage. Sensory function in the foreskin and glans remained preserved.

The patient disclosed that the metallic ring was a component of an automobile engine. Its composition was thought to be steel, although the precise composition of the material remained undetermined. Initial attempts to sever the ring using a standard ring cutter and rib shears proved futile, achieving minimal surface impact. Subsequently, it was suggested that an air cutter, a device frequently used by rescue teams, might be effective in cutting through the ring. This led to the summoning of a rescue unit. Upon their arrival, the rescue team immediately began the cutting operation. Sedation was administered to the patient for the duration of the procedure.

Taking into account the limited clearance between the metal ring and the penile tissue, meticulous precautions were taken to prevent skin damage. Forceps were utilized to gently pull and stabilize the ring away from the skin. Additionally, a surgical brain spatula was carefully inserted into the narrow space between the ring and the penis, serving as a protective barrier against the skin.

**Figure 2:**
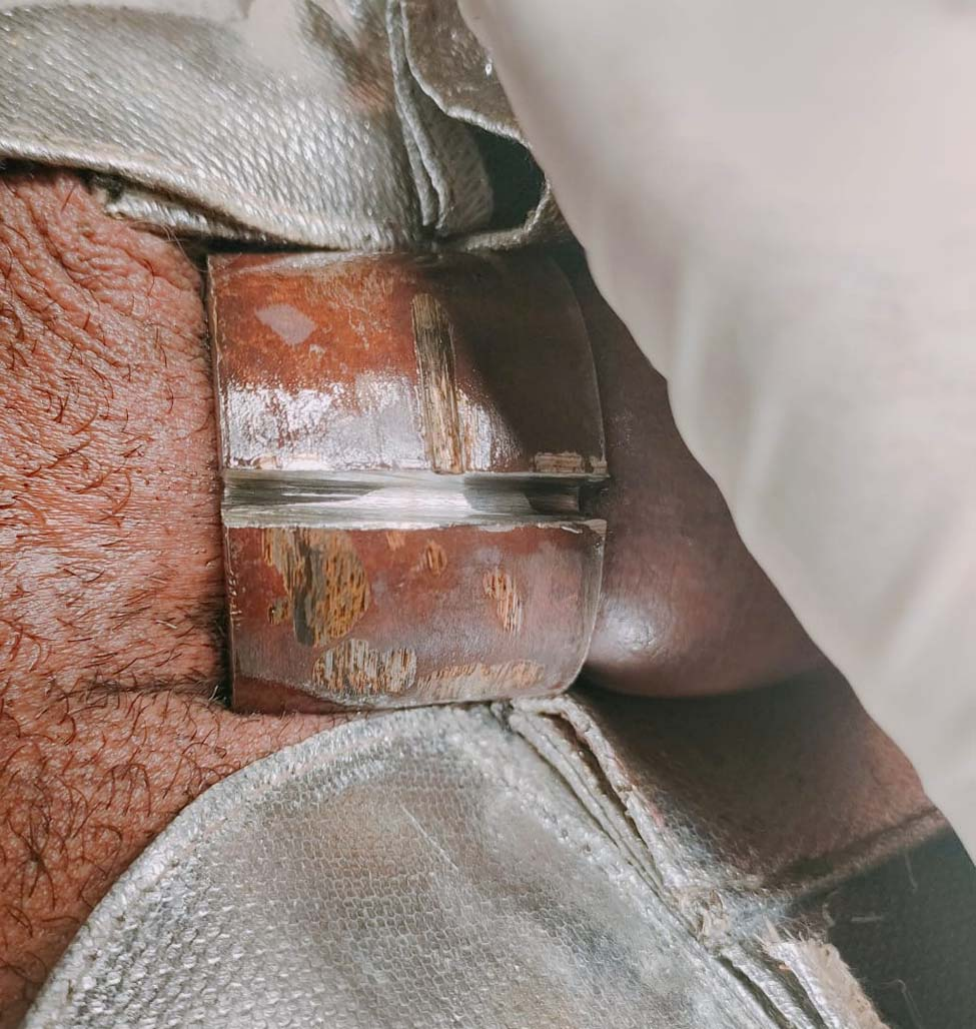
‘Metal ring during the removal procedure’. This figure provides a detailed view of the metal ring’s hardness and thickness captured in the mid-cutting process. Illustrating the challenges faced in severing the ring due to its robust material, it requires specialized cutting tools.

The metal ring was notably robust (Fig. 2), necessitating two distinct cuts at the 12 o’clock and 6 o’clock positions for removal. During the first incision, the rescue team positioned itself on the patient’s right, aligning the air cutter blade toward the head, where more space was available. For the second incision, the team repositioned to the left of the patient, angling the blade in the direction of the feet. Extra caution was taken to shield the distal portion of the penis with gauze throughout the cutting process.

The placement of the surgical brain spatula between the penile skin and the metal ring was initially challenging due to the proximity to the pubic bone and the constrained angle. However, this task was facilitated after the first cut was completed, which increased the mobility of the ring. After the ring was removed, a urinary catheter was placed as a precautionary measure.

**Figure 3:**
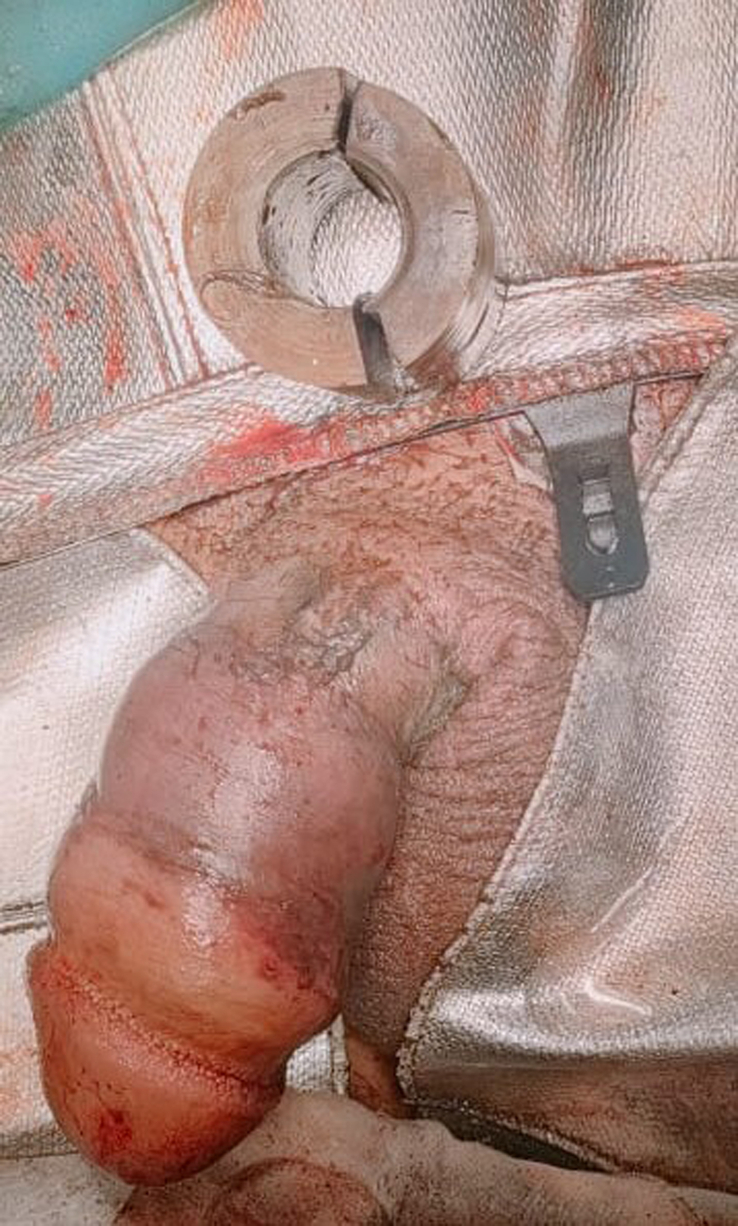
‘Postoperative recovery’. This postoperative image depicts the penis immediately after removal of the ring, cut into two pieces. It highlights the rapid reduction in swelling and the beginning of normal recoloration, indicating the restoration of blood flow.

The total duration from the patient’s emergency transport to the arrival of the rescue team spanned ~2 h. The constriction was successfully alleviated around 90 min following their arrival. In particular, swelling and discoloration began to subside immediately after the cuts were made (Fig. 3). Six hours after the procedure, a marked improvement in penile coloration was observed, and swelling was significantly reduced within 2 days. The urinary catheter was removed the day after the constriction was relieved. The patient was discharged on the fourth day after the procedure, following confirmation of normal urination.

During a follow-up examination 10 days after the incident, partial skin sloughing was observed at the base of the penis. However, it was in the process of crusting over. Importantly, the patient did not show abnormal urination at this stage.

## Discussion

### Epidemiology and classification of penile strangulation cases

Penile strangulation, a medical emergency, occurs when a foreign object compresses and encircles the entire circumference of the penis, disrupting circulation. This disruption can lead to edema and swelling distal to the construction site, with a potential risk of necrosis in the affected area, underscoring the urgency of timely intervention.

Objects that cause penile constriction can be classified into hard materials, such as metal rings and plastic bottles, and soft materials such as rubber bands, threads, and strings^1,3,4^. The removal of soft constraining objects often requires more time, and a higher incidence of serious complications is observed, including necrosis^5,6^. This is attributed to their smaller surface area of constriction, intense pressure due to contraction, and often delayed identification and extended duration of constriction, as they can become embedded in the tissue. While hard constricting objects are generally associated with fewer complications, the risk increases significantly if the duration of the constriction exceeds 6 h^3^. Therefore, prompt release of the constriction is critical for favorable outcomes.

### Methodologies for the removal of constricting objects

There are occasional reports that advocate the use of dental air turbines, commonly used in oral surgery, to remove hard-constricting objects^7^. Their effectiveness is attributed to the reduced time required for cutting. However, the literature on the specific duration needed for such procedures remains scarce.

Research by Zhu and Wang provides valuable information on the methodologies and timeframes necessary to manage penile strangulation cases^7^. Their analysis reveals that the average duration for cutting through hard constricting objects using a dental air turbine, in nine cases, was ~65 min. This contrasts with two other instances where air cutters were used for metal objects resistant to dental air turbines, and these cases recorded significantly shorter cutting times of 8 and 10 min, respectively^8^. Such findings suggest a marked efficiency of air cutters in these scenarios.

However, it is important to note the challenges in making direct comparisons between different cases. Variability in the type, size, and thickness of the constricting objects can significantly affect the choice of tool and the time required for successful removal. Despite these variables, air cutters have emerged as particularly effective in situations involving more challenging constricting objects, as evidenced by the shorter cutting times observed in the aforementioned cases^7^.

### Case-specific challenges and solution approaches

In the case we report, the duration of the cutting procedure extended to 90 min, which is notably longer compared to the average times reported by Zhu and Wang^7^. This extended duration can be attributed to several specific factors. Firstly, the metal’s thickness and hardness posed a significant challenge. Secondly, the severe edema that exists reduced the available space between the penile skin and the constricting ring, complicating the cutting process. Additionally, there was an operational delay due to the need to replace the air cylinder mid-procedure, a consequence of the air cutter’s high consumption of compressed air.

Taking into account these unique circumstances, it is unlikely that the use of a dental air turbine, typically faster in other contexts, would have expedited the release of the constriction in this particular case. The specific challenges encountered underscore the importance of selecting the appropriate tool based on the individual characteristics of each case, rather than relying solely on the average cutting times reported in the literature.

In the case we presented, the insertion of a surgical brain spatula between the penile skin and the metal ring was a critical precautionary measure to minimize the risk of skin damage during the cutting procedure. Zhang *et al*.^9^ have also emphasized the importance of seeking help from external agencies, such as rescue teams, in cases of penile strangulation involving materials such as steel rings. This recommendation arises from the fact that emergency rooms and outpatient clinics are often not equipped with the specialized tools required for such procedures.

Furthermore, it is crucial to recognize that cutting tools, although not classified as medical devices, require the handling of individuals who are not only trained, but also experienced in their use. This ensures that the procedures are carried out safely and effectively. Safety considerations during the cutting process are of utmost importance, given the delicate nature of the task and the potential risks involved.

### Role of multidisciplinary collaboration and external assistance

The successful resolution of this complex case of penile strangulation underscores the critical role of interdisciplinary teamwork and the integration of external resources. The participation of a specialized rescue unit alongside our medical team was instrumental in effectively managing the emergency, highlighting several critical aspects of the management of unusual and challenging medical situations.

In cases such as penile strangulation, where conventional medical tools and expertise may be inadequate, the necessity for interdisciplinary collaboration becomes paramount. The diverse skill sets and knowledge bases of different professionals, including urologists, emergency physicians, and external rescue teams, contribute significantly to the overall success of the procedure. This collaboration not only brings together diverse perspectives, but also facilitates the sharing of unique skills and techniques that are vital in emergency situations.

The participation of external agencies, particularly those equipped with specialized tools not typically found in medical settings, can be a deciding factor in the successful resolution of such cases. As demonstrated in our case, the use of an air cutter – a tool more commonly associated with rescue operations than medical procedures – was crucial to safely and efficiently remove the metallic ring. This collaboration emphasizes the need for medical teams to be adaptable and resourceful, willing to seek and use non-conventional resources when faced with atypical clinical challenges.

### Educational outreach and prevention

The incident described in our report highlights the necessity of comprehensive sexual health education, particularly regarding the risks associated with using nonmedical objects for sexual activities. It is imperative that healthcare professionals actively participate in public awareness campaigns that highlight the potential dangers and medical complications that can arise from such practices.

Urologists, emergency medicine specialists, and other healthcare providers play a crucial role in preventive health education. They should offer clear guidance and advice on the hazards of misusing inappropriate objects during sexual activities. Utilizing educational materials and counseling can be effective in disseminating this vital information.

Additionally, an essential aspect of prevention is to educate the public about the urgency of seeking immediate medical attention for events involving genital injury or abnormalities. Initiatives to destigmatize these incidents are crucial, as they encourage timely medical intervention, which can prevent severe complications.

Healthcare professionals should also be reminded of the importance of a nonjudgmental and empathetic approach when handling sensitive cases. A supportive and respectful treatment environment is key to foster open communication with patients, helping to ensure effective management and educational efforts.

In light of our findings, there is a clear imperative for enhanced educational outreach. Sensitivity to safe practices in sexual health and the critical need for immediate medical care in emergencies are vital. This approach not only contributes to better patient outcomes, but also supports broader public health objectives.

## Conclusion

This case report presents a particularly challenging incident of penile strangulation induced by a metallic ring. The resolution of this case was based on the synergistic efforts of our medical team in conjunction with a specialized rescue unit, showcasing the indispensable role of multidisciplinary teamwork in handling such complex medical emergencies.

Our report contributes significantly to the existing literature on penile strangulation by illustrating the necessity for innovative approaches in emergency medical procedures. In this case, the use of specialized tools such as air cutter and the strategic use of a surgical brain spatula were instrumental and highlighted the need for improvisation and adaptability in medical emergencies. Such interventions were crucial in mitigating further harm and assuring patient safety throughout the complex removal procedure.

Furthermore, this case underscores the importance of preparedness for rare medical scenarios and the need for protocols that allow rapid coordination with external agencies for specialized assistance. This experience suggests that the integration of nontraditional medical tools and interdisciplinary collaboration can greatly improve outcomes in similar complex cases.

In light of this case, there is an obvious need for ongoing research on the management of rare and acute urological emergencies. Our findings indicate that greater awareness among medical professionals about such atypical emergencies could lead to better preparedness and more effective response strategies. Furthermore, this report calls attention to the potential to incorporate unconventional techniques and tools into medical practice to better handle unforeseen clinical challenges.

Lastly, this report not only adds valuable information to the limited yet essential literature on penile strangulation, but also invites future discussions on innovative emergency interventions, interdisciplinary collaboration, and readiness for unique medical emergencies.

## Ethical approval

Not applicable.

## Consent

Written informed consent was obtained from the patient for the publication of this case report and any accompanying images. A copy of the written consent is available for review by the Editor-in-Chief of this journal upon request.

## Sources of funding

Not applicable.

## Author contribution

All authors have contributed equally to the work reported in this manuscript, including the conception, design, execution, data acquisition, analysis and interpretation, and the drafting and revising of the manuscript for important intellectual content.

## Conflicts of interest disclosure

The authors declare that they have no conflicts of interest concerning this article.

## SCARE Guidelines

The work has been reported according to the SCARE criteria^10^.

## Research registration unique identifying number (UIN)

Not applicable.

## Guarantor

Wael Gazzah.

## Data availability statement

The datasets are available from the corresponding author on reasonable request.

## Provenance and peer review

Not commissioned, externally peer-reviewed.
